# Rising prevalence of subthreshold and major depressive symptom in South Korea: A trend analysis from 2014 and 2018

**DOI:** 10.1371/journal.pone.0320980

**Published:** 2025-04-21

**Authors:** Djoko Priyono, Sanghee Kim

**Affiliations:** 1 College of Nursing, Keimyung University, Daegu, Republic of Korea; 2 School of Nursing, Faculty of Medicine, Tanjungpura University, Pontianak, West Kalimantan, Indonesia; University of Cologne: Universitat zu Koln, GERMANY

## Abstract

**Introduction:**

Subthreshold depression and major depressive symptoms are prevalent mental health conditions that significantly impact quality of life and contribute to South Korea’s high suicide rate. Despite their importance, few studies have examined temporal changes in the occurrence of these disorders in the Korean population.

**Objective:**

This study aimed to estimate the prevalence of subthreshold depression and MDD using a large, representative sample of the South Korean population and analyze trends over time.

**Methods:**

Data were obtained from 10,848 participants aged 19 and above in the Korean National Health and Nutrition Examination Survey (KNHNES) in 2014 and 2018. Depression severity was categorized using cutoff scores of 5-14 for subthreshold symptoms and ≥ 15 for severe symptoms.

**Results:**

The prevalence of subthreshold depression increased from 12.90% in 2014 to 15.20% in 2018, while MDD rose from 4.7% to 7.0% (p < 0.001). Logistic regression analysis revealed that MDD (OR = 8.1, 95% CI = 2.34-11.23), college education (OR = 7.9, 95% CI = 4.23-10.00), and age above 65 years (OR = 8.1, 95% CI = 2.58-12.58) exhibited similar risks for suicide attempts.

**Conclusion:**

Since 2014, there has been a sharp and sustained increase in both subthreshold and severe depressive symptoms among the Korean population. This alarming trend underscores the critical need for targeted prevention and intervention strategies.

## Introduction

Depression is a significant global health issue, impacting around 280 million people worldwide. According to the World Health Organization, depression is a major contributing factor to both disability and the total burden of disease worldwide [1]. This disorder is characterized by long-lasting feelings of sadness, a lack of interest in daily activities, and various cognitive and physical symptoms that significantly impair an individual’s ability to function in daily life [2].

The prevalence of depression continues to increase globally. In 2020, depression ranked second among all causes of disability worldwide. According to the Global Burden of Disease Study, roughly 7% of individuals over 60 years old exhibit clinically depressive symptoms [3]. However, this estimate likely underestimates the actual prevalence, especially among older age groups. If individuals with mild or subthreshold depression—wherein symptoms are not severe enough to meet the criteria for clinical depression—are included, the prevalence significantly rises [4,5]. Recent research suggests that subthreshold depression affects approximately 10% of older adults in the general population and up to 25% of those in primary care settings [6]. This indicates that, despite not meeting the full clinical criteria, subthreshold depression has serious consequences, including increased functional impairment, reduced quality of life, and exacerbated existing health conditions [7]. Additionally, subthreshold depression often precedes more severe major depression, making early identification and intervention vital for preventing further complications [8].

In South Korea, depression affects people’s well-being and contributes to the country’s high suicide rate, raising serious public health concerns [9]. The prevalence of major depression in South Korea is approximately between 3.5-4%, with higher rates observed among lower-income populations [10]. In contrast, Japan has a relatively lower prevalence of depression, with studies indicating that about 1.2% of the population experiences major depressive disorders [11]. The Organization for Economic Cooperation and Development (OECD), an international organization comprising 38 member countries representing the world’s most advanced economies, reports that the suicide rate in South Korea is more than twice the average rate across OECD countries, with 25.2 out of every 100,000 people in 2022. This figure remains significantly higher than the prevalence of suicide in Japan, which stands at 16.5 out of every 100,000 people as of 2018. Despite both nations implementing various suicide prevention measures, South Korea continues to have the highest suicide rate among OECD countries, while Japan has seen a notable decrease from its peak of 25.7 per 100,000 in 2009 [12–14].

The high rate of major depression in South Korea was influenced by various factors, including cultural aspects and policies aimed at addressing depression. The rigorous education system pressures students to achieve academically from a young age [15]. Additionally, high living costs and substandard housing conditions contribute to the prevalence of depression [16]. In workplaces, hierarchical environments pressure employees to perform, with a fear of repercussions from superiors and expectations to work overtime without compensation. This work culture discourages open discussions about mental health and perpetuates the stigma surrounding seeking help, leaving many individuals feeling isolated and unsupported in their struggles with depression [17,18]. Moreover, mental health policies in South Korea have not yet proven effective in reducing depression rates. Psychological support services are often not tailored to specific demographic characteristics such as age, socio-economic status, or regional context, and they are insufficiently research-based to address these issues [19]. Furthermore, South Korea’s prescription rate for antidepressants is significantly lower than the OECD average, with only 19.9 defined daily doses per 1,000 inhabitants per day as of 2016 [20]. This low prescription rate indicates that depression remains under-treated despite its high prevalence, contributing to South Korea having the highest suicide rate among OECD countries [21].

Despite increasing interest in tracking changes in the prevalence of depression over time, existing studies have produced conflicting results. A meta-analysis using age-adjusted estimates revealed no significant change, despite some studies showing an increase in prevalence [22]. This uncertainty highlights the difficulties in assessing long-term trends, mainly due to variations in measures and methodologies used across studies. Sociodemographic factors such as sex, age, employment status, marital status, educational attainment, and income are known to influence the prevalence of depression, with higher rates typically found in women, individuals living alone, unmarried people, unemployed individuals, and individuals with low educational attainment and income [23,24]. Nevertheless, data on how the prevalence of depression changes over time within these sociodemographic subgroups are limited [25].

Given the influence of sociocultural context on the prevalence of depression, it is crucial to evaluate the epidemiology of subthreshold and severe depression across different cultural settings. To close this gap, this study aimed to determine how the prevalence of subthreshold and severe depression changed over time across a wide range of sociodemographic groups in South Korea using a large national sample. By analyzing data from surveys conducted in 2014 and 2018, this study is expected to offer a comprehensive overview of depression trends in the country over time.

## Materials and methods

### Data collection

Data were collected from the Korea National Health and Nutrition Examination Survey (KNHANES) conducted from 2014 and 2018. The KNHANES is a comprehensive, publicly conducted survey targeting a multi-stage clustered sample from the non-institutionalized demographic of South Korea [26]. This survey encompasses health-related interviews, nutritional assessments, and medical examinations. Data were acquired through household visits, during which standardized physical check-ups were performed in mobile examination facilities. A team comprising physicians, nurses, and community volunteers conducted in-person interviews and physical assessments using these mobile units. Primary sampling units were selected, and survey weights were assigned to adjust for the sophisticated survey structure and post-stratification. From 2014 and 2018, 15,742 individuals were chosen for the survey. Those aged 19 years and above (n =  10,848) were selected for this research after the exclusion of incomplete data.

### Dependent variables

Depression was evaluated using the Patient Health Questionnaire-9 (PHQ-9). The PHQ-9 is an established medical tool used to diagnose, monitor, and assess the intensity of depression [27]. The Korean version of the PHQ-9, which was utilized in the 2016 KNHANES, showed excellent internal consistency (r =  0.88) and long-term reliability (r =  0.60), especially among older adults [28]. The PHQ-9 has nine criteria that indicate the frequency of depressive symptoms experienced throughout the previous 2 weeks. Every criterion is accompanied by four response choices: “not at all,” “several days,” “more than half the days,” and “nearly every day,” corresponding to scores of 0, 1, 2, and 3, respectively. The overall scores range from 0 to 27, with a higher score indicating a higher severity of depression [29].

Previous studies have determined the PHQ-9 cutoff scores for reliably categorizing the severity of depression. Han et al., (2008) found that a PHQ-9 cutoff score of 5 had a 0.78 specificity and 0.80 sensitivity for diagnosing depression. Cutoff scores of 5, 10, 15, and 20 were established to differentiate between moderate, severe, mild, and moderately severe depression, respectively [30]. With cutoff scores ranging from 8 to 11, a meta-analysis validated the PHQ-9’s adequate diagnostic accuracy for major depressive disorder. The majority of the 18 validation studies reviewed recommended a cutoff score of 10 [31]. Consequently, participants were divided into three groups: those with PHQ-9 scores of 0–4 indicated no/minimal depressive symptoms, 5–14 identified subthreshold symptoms, and 15 and higher indicated severe symptoms.

To examine suicidal attempts, participants were asked to indicate whether they had made a suicide attempt in the previous 12 months. The question was: “Have you attempted suicide during the past year? “Have you attempted suicide during the past year?” Respondents could choose between “yes” or “no.”

### Independent variables

Numerous variables were gathered through health-related interviews and examination questionnaires. Age was classified into five groups: 19–29, 30–49, 50–59, 60–64, and ≥ 65 years. Socioeconomic status was measured based on household income and educational attainment. Monthly income was adjusted for family size (monthly income/ √ number of family members) and divided into quartiles: lowest, lower-middle, upper-middle, and highest. Educational attainment was categorized based on years of schooling: elementary school or lower, middle school, high school, and college or higher. Lifetime alcohol consumption was classified as either “never” or “yes.” For individuals who answered affirmatively, additional information was gathered regarding the age at first full drink and the frequency of drinking in the past year, categorized as “not at all,” “less than once a month,” “once a month,” “two to four times a month,” “two to three times a week,” and “four or more times a week.” Smoking status was divided into three groups: never smokers, former smokers, and current smokers. Never smokers included those who had either never smoked or smoked less than 100 cigarettes in their lifetime. Individuals who had smoked more than 100 cigarettes were classified as smokers. Among them, those who were actively smoking were classified as current smokers, while those who were no longer smoking were classified as former smokers, regardless of when they had stopped smoking.

### Ethical issues

Ethical clearance was unnecessary for this study due to the public availability of the KNHANES 2014 and 2018 survey data (http://knhanes.cdc.go.kr/knhanes/ accessed July 18, 2024). Before the survey, all participants were notified that they had been selected randomly to participate in the KNHANES survey and had the option to decline involvement in subsequent analysis. Written informed consents were then acquired. Complete anonymization was applied to the data obtained from the KNHANES database.

### Statistical analysis

Participant characteristics were presented and analyzed as frequencies and percentages. All estimates and 95% confidence intervals (CIs) were adjusted to ensure national representativeness. The prevalence of subthreshold and severe depression was analyzed based on age and sex. The odds ratio (OR) for subthreshold and severe depression in 2014 and 2018 was calculated using logistic regression based on the depression status, stratified by the sociodemographic category. Additional sociodemographic variables were accounted for as covariates in the logistic regression analysis of each sociodemographic variable to minimize their influence as possible confounding factors. The analysis of the main and interaction effects of sociodemographic factors utilized a Generalized Linear Model (GLM) with a univariate approach, using depression as the dependent variable. The independent variables included age, gender, education, and income. We employed a full factorial design and applied Type III sums of squares. This analysis allowed us to verify the main effect of each variable and examine the interaction effects by combining two variables, such as age x gender.

## Results

A total of 10,848 participants who completed the KNHANES in 2014 and 2018 were included in this study. Table 1 shows the sociodemographic characteristics of the participants. The participant characteristics exhibited specific variations between 2014 and 2018. According to sex, the distribution of age and educational attainment showed a continuing upward trend. Only the female participants who completed elementary school exhibited a decline between 2014 and 2018. There was an upward trend in the distribution of the lowest income for both male and female groups in 2014 and 2018. The proportion of current smokers exhibited regular and progressive fluctuations over the years. In addition, the per-capita drinking frequency in the once-a-month group among the women decreased from 11% to 9.3%. Conversely, the proportion of women who reported alcohol consumption two to three times per week substantially increased (from 8.75% to 10.3%). (Table 1)

**Table 1:** Sample characteristics of KNHANES 2014 and 2019.

Characteristics	Men		Women	
**2014** **N = 2050**	**2018** **N = 2586**	**2014** **N = 2880**	**2018** **N = 3332**
Age group (years)				
19 – 29	232 (11.3)	350 (13.5)	344 (11.9)	393 (11.9)
30 – 49	703 (34.3)	859 (33.2)	1004 (34.9)	1095 (33.0)
50 – 59	386 (18.8)	469 (18.1)	570 (19.8)	667 (20.1)
60 – 64	174 (8.5)	272 (10.5)	256 (8.9)	313 (9.4)
65 Years older	555 (27.1)	636 (24.6)	706 (24.5)	848 (25.6)
Education				
Elementary school or lower	343 (16.7)	335 (13.0)	829 (28.8)	792 (23.8)
Middle school	249 (12.1)	250 (9.7)	291 (10.1)	345 (10.4)
High school	718 (35.0)	945 (36.5)	913 (31.7)	1039 (31.2)
College graduate or higher	740 (36.1)	1055 (40.8)	847 (29.4)	1156 (34.7)
Income				
Lowest	486 (23.7)	626 (24.2)	690 (24.0)	818 (24.5)
Lower middle	532 (26.0)	652 (25.2)	728 (25.3)	947 (25.4)
Upper middle	515 (25.1)	660 (25.5)	736 (25.6)	823 (24.7)
Highest	517 (25.2)	642 (24.8)	726 (25.2)	834 (25.0)
Smoking status				
Current smoker	811 (39.2)	876 (34.0)	136 (4.7)	198 (6.0)
Ex smoker	779 (37.7)	1094 (42.4)	159 (5.4)	203 (6.1)
Never smoking	477 (23.1)	608 (23.6)	2624 (89.9)	2915 (87.9)
Drinking Frequency				
Never	403 (19.5)	437 (17.0)	1102 (37.8)	1143 (34.5)
Less than once a month	205 (9.9)	346 (13.4)	690 (23.6)	783 (23.6)
Once a month	187 (9.0)	204 (7.9)	320 (11.0)	308 (9.3)
1–4 times per month	533 (25.8)	685 (26.6)	479 (16.4)	656 (19.8)
2–3 times per week	456 (22.1)	580 (22.5)	254 (8.7)	340 (10.3)
4 or more times a week	283 (13.7)	326 (12.6)	74 (2.5)	86 (2.6)

Values are presented as number (%)

**Fig 1:**
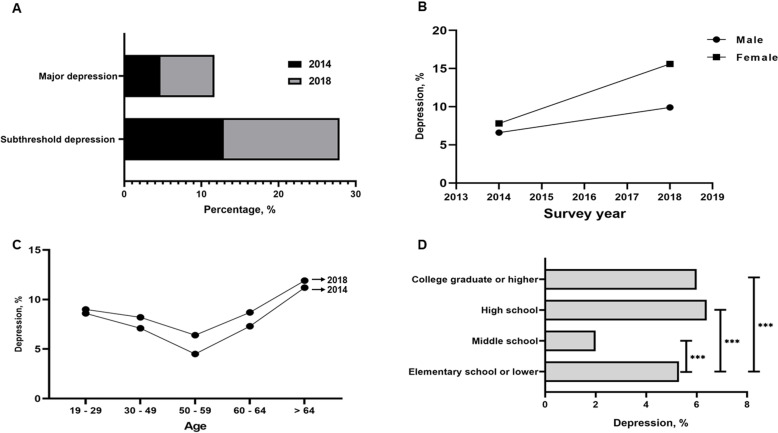
Prevalence depression. A, Increasing prevalence subthreshold depression and major depression from 2014 and 2018. B, Increasing prevalence depression based on gender difference. C, Increasing prevalence depression based on age difference. D, Difference prevalence of depression based on education level.

**Fig 1** presents the prevalence of depression between 2014 and 2018. The data revealed two significant trends. First, the prevalence of subthreshold depression increased from 12.90% in 2014 to 15.20% in 2018. Second, the prevalence of major depression rose from 4.7% in 2014 to 7.0% in 2018 (*p* <  0.001). These findings indicated a notable increase in the prevalence of both subthreshold and major depression over 4 years. Although the prevalence of depression among both women and man increased between 2014 and 2018, the prevalence remains higher among women. Among adults aged 65 and older, depression was most common in both 2014 and 2018. However, the most notable increase in depression prevalence occurred in the 50-59 age group. On the other hand, the highest prevalence was found among high school graduates, followed by those with a college degree or higher.

The prevalence of subthreshold depression according to the sociodemographic variables was also analyzed. From 2014 and 2018, the prevalence of subthreshold depression among both men and women continued to rise significantly. In 2014, subthreshold depression was associated with the lowest income level, drinking frequency of two to four times per week, and drinking frequency of more than four times per week among the women. The same results were observed in 2018, wherein female sex, drinking frequency of two to three times per week, and drinking frequency of more than four times per week were significant predictors of subthreshold depression. However, upper-middle monthly income and former smoking were also associated with subthreshold depression (Tables 2 and 3).

**Table 2:** Time trends in subthreshold depression 2014 vs 2018.

Characteristics	Prevalence		AOC (95%)	
2014	2018	2014	2018
Gender				
Male	4.2	4.9	0.57 (0.48-0.67)	0.63(0.58-0.76)
Female	8.7	10.4	1.73 (1.47-2.05)^**^	1.78(1.51-2.09)^***^
Education				
Elementary school or lower	4.2	2.6	1.40(1.14-1.72)	1.65(0.81-3.24)
Middle school	1.5	1.3	0.69(0.52-0.93)	0.87(0.65-1.18)
High school	4.3	5.1	0.74(0.60-0.91)	0.95(0.77-1.18)
College graduate or higher	4.1	4.8	0.71(0.57-0.87)	0.82(0.66-1.02)
Income				
Lowest	3.9	4.3	1.47(1.17-1.85)^***^	0.85(0.69-1.04)
Lower middle	3.6	4.2	1.26(1.01-1.58)	0.68(0.54-0.84)
Upper middle	3.0	3.3	0.95(0.75-1.21)	0.52(0.41-0.65)^**^
Highest	2.4	3.4	0.20(0.16-0.26)	0.11(0.09-0.13)
Smoking status				
Current smokier	2.6	3.2	2.03(1.21-6.03)	1.55(0.71-2.21)
Ex smoker	2.2	2.3	1.07(0.86-1.33)	0.63(0.43-0.91)^***^
Never smoking	8.2	9.9	0.72(0.57-0.90)	0.66(0.49-0.89)
Drinking Frequency				
Never	4.4	3.6	1.77(0.94-5.01)	1.52(0.73(2.71)
Less than once a month	2.4	3.0	1.08(0.86-1.36)	0.91(0.72-1.14)
Once a month	1.2	1.6	0.98(0.74-1.31)	0.97(0.72-1.30)
1-4 times per month	2.9	2.9	0.91(0.72-1.14)	0.90(0.72-1.12)
2-3 times per week	1.9	2.3	1.03(0.80-1.32)^*^	0.86(0.67-1.10)^**^
4 or more times a week	1.0	1.1	1.00(0.72-1.39)^**^	1.06(0.77-1.46)^**^

AOR =  adjusted odds ratio, CI =  confidence interval. a %: weighted by age and sex to the population census for each year; b Adjusted for other sociodemographic factors in multiple logistic regression analyses using the enter method.

* P <  0.05;

** P <  0.01;

*** P <  0.001.

**Table 3:** Time trends in Major Depression 2014 vs 2018.

Characteristics	Prevalence		AOC (95%)	
2014	2018	2014	2018
Gender				
Male	6.6	7.8	0.40(0.31-0.51)	0.45(0.34-0.59)^***^
Female	9.9	15.6	2.47(1.92-3.19)^***^	2.20(1.68-2.88)^***^
Education				
Elementary school or lower	2.7	1.4	1.04(0.85-1.27)	
Middle school	0.7	0.5	0.51(0.34-0.75)	0.70(0.46-1.08)
High school	1.5	2.0	0.48(0.36-0.63)	0.57(0.42-0.78)
College graduate or higher	1.3	1.7	0.37(0.28-0.50)	0.44(0.32-0.61)
Income				
Lowest	2.7	3.1	0.85(0.69-1.06)^***^	0.82(0.69-1.04)
Lower middle	1.8	2.0	0.57(0.43-0.76)^**^	0.68(0.54-0.84)
Upper middle	1.5	1.2	0.45(0.33-0.61)^*^	0.52(0.41-0.65)
Highest	1.1	0.8	0.32(0.23-0.45)^*^	0.11(0.09-0.13)
Smoking status				
Current smoker	1.5	1.2	1.02(0.71-4.31)	1.54(0.97-7.11)
Ex smoker	0.9	1.0	0.50(0.34-0.74)^***^	0.63(0.43-0.91)^*^
Never smoking	4.6	2.6	0.88(0.67-1.16)	0.66(0.49-0.89)^**^
Drinking Frequency				
Never	2.8	0.8		
Less than once a month	1.1	0.3	0.61(0.43-0.85)^*^	0.67(0.47-0.96)^*^
Once a month	0.6	0.8	0.58(0.38-0.89)	0.59(0.35-0.98)^*^
1-4 times per month	1.1	1.6	0.54(0.39-0.76)^***^	0.53(0.35-0.98)^***^
2-3 times per week	0.7	0.8	0.52(0.35-0.76)	0.70(0.48-1.03)
4 or more times a week	0.7	0.9	0.99(0.66-1.48)^**^	1.13(0.73-1.76)^**^

AOR =  adjusted odds ratio, CI =  confidence interval. a %: weighted by age and sex to the population census for each year; b Adjusted for other sociodemographic factors in multiple logistic regression analyses using the enter method.

* P <  0.05;

** P <  0.01;

*** P <  0.001.

**Fig 2:**
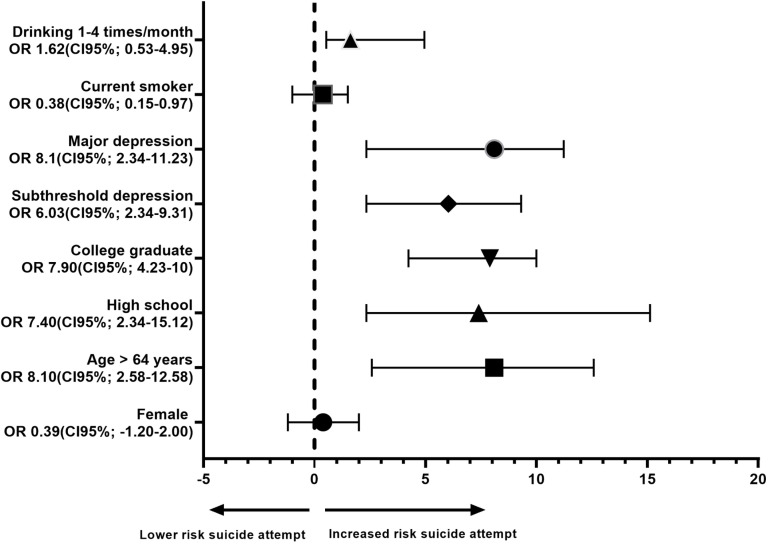
Risk for a suicide.

**Fig 2** shows the risk for a suicide attempt according to different characteristics including subthreshold depression, major depression, age, sex, educational attainment, drinking status, and smoking status. Major depression (OR =  8.1, 95% CI =  2.34–11.23), college education (OR =  7.9, 95% CI =  4.23–10.00), and age above 65 years (OR =  8.1, 95% CI =  2.58–12.58) exhibited an almost equal risk for a suicide attempt. Another factor associated with this risk was drinking status, with the participants consuming alcohol one to four times per month having a higher risk (OR =  1.62, 95% CI =  0.53–4.95) than the current smokers and women.

The main effects of age, gender, education, and income on depression are presented in **Table 4**. It was found that age and education did not have significant main effects on depression. However, significant interaction effects were identified among age x gender, age x income, gender x income, and education x income. While age and education alone did not show significant effects, the interactions between age x gender (p <  0.05) and age x income (p <  0.001) were significant.

**Table 4.** Main and interaction effect of socio-demographic characteristics.

Category	*SS*	*df*	*F*	Partial Eta Squared
Age	0.005	1	0.125	0.016
Gender	0.203	1	4.606^**^	6.122
Education	0.008	1	0.198	1.021
Income	0.287	1	6.504^*^	2.781
Age x gender	0.002	1	0.630^*^	1.300
Age x education	0.052	1	1.057	1.428
Age x income	0.012	1	0.285***	1.371
Gender x education	0.051	1	1.168	5.513
Gender x income	0.058	1	1.328^*^	5.213
Education x income	0.011	1	0.264^**^	1.255

SS = Sum of Square,

* P < 0.05;

** P < 0.01;

** *P < 0.001

## Discussion

This study examined the prevalence and trends of subthreshold and major depression across sociodemographic subgroups stratified by sex using nationally representative data from the South Korean adult population in 2014 and 2018. The findings revealed a steady increase in the prevalence of subthreshold and major depressive symptoms, with variations across different sociodemographic groups. The most significant increase in the prevalence of subthreshold depression was observed among the men and women, middle and high school graduates, college graduates, and individuals consuming alcohol four or more times per week. The prevalence of major depressive symptoms showed the most significant increase among the women, individuals with lower and upper-middle income levels, former smokers, and individuals drinking two to three times per week and four or more times per week. Additionally, our study identified major depression, age over 65 years, and college education as high-risk factors for suicide attempts, overshadowing other variables such as drinking frequency, smoking status, and sex.

Between the 2014 and 2018 surveys, there were significant differences in several sociodemographic factors, with a particular emphasis on the rising number of individuals over the age of 60 years. This change is indicative of the broader demographic trends in South Korea, which are characterized by an aging population due to a declining birth rate and an increasing lifetime expectancy [32]. The proportion of individuals aged 60–64 years increased from 8.5% to 10.5% among the men and from 8.9% to 9.4% among the women in 2018, mirroring the country’s demographic transition. Years of consistently low fertility rates have reduced the younger population, while advancements in healthcare have led to increased longevity, creating a rapidly aging society [33].

The likelihood of developing depression is significantly higher when individuals perceive their health status and stress level as poor. Research conducted in Korea has demonstrated a strong correlation between self-rated health and the incidence of depression. Similar findings have been observed in studies of older adults in other nations [34]. Furthermore, a recent study uncovered a strong link between perceived stress and depression, which aligns with previous reports. Notably, this study’s limitations included the lack of evaluation of perceived stress using a validated assessment tool [35].

Although the prevalence of subthreshold and major depression increased in South Korea from 2014 to 2018, it remains lower than that reported in the United States (9.7% based on 2015–2020 surveys) [36]. This result is consistent with other reports suggesting that depression is far less common in Asian countries than in Western and Middle Eastern countries [34]. Nevertheless, the data obtained from the KNHANES depended only on a basic questionnaire instead of comprehensive face-to-face interviews, potentially explaining the lower reported rates. Studies have shown that self-reported questionnaires can yield higher prevalence estimates due to sociocultural factors influencing the perception and reporting of depressive symptoms [37].

Consistent with prior research, our study demonstrated a gradual rise in the prevalence of depression over time, with a notable increase in that of major depressive disorder within the South Korean population from 4.7% in 2014 to 7.0% in 2018. This increase surpasses the growth rate observed in the United States during the same period, suggesting that South Korea has achieved remarkable success in recent decades in combining rapid economic growth with the gross domestic product (GDP) growing on average by 5.7% annually between 1980 and 2023 [38]. Over recent decades, South Korea has experienced rapid economic development and cultural transformation, heightening societal competition and stressors such as rising housing costs, job insecurity, and divorce rates—all linked to increased depressive symptoms [39].

The present study also found that the prevalence of depression among the high school and college graduates increased more significantly than that among the other sociodemographic groups between 2014 and 2018. Academic and career pressures, particularly among South Korean students, contribute substantially to this trend. The country’s highly competitive educational system, high tuition fees, and challenging job market create a stressful environment for students and recent graduates [40,41]. The high cost of education, second only to that in the United States among OECD countries, and the increasing rate of youth unemployment exacerbate mental health challenges within this demographic [42].

Among our female participants, the prevalence of subthreshold and major depression increased significantly, highlighting the unique intersection of traditional gender expectations and modern societal pressures faced by South Korean women [43]. Despite progress in gender equality, cultural norms continue to place disproportionate caregiving and household responsibilities on women, contributing to conflicts in work–life balance and chronic stress [44]. Additionally, women are more vulnerable to workplace discrimination, sexual harassment, and wage disparities, further compounding their mental health burden [45].

Regarding alcohol use, the study found an increased prevalence of depression among both men and women, with varying drinking frequencies noted from 2014 and 2018. Alcohol consumption is deeply rooted in South Korean business and social culture, and its prevalence has increased in recent decades due to societal stressors and cultural norms [46]. The bidirectional relationship between alcohol use and depression often creates a self-perpetuating cycle, wherein individuals with depression may turn to alcohol for temporary relief. In contrast, chronic alcohol use can exacerbate or trigger depressive symptoms through neurochemical changes and social consequences [47].

This study also highlighted a stark disparity in the cumulative prevalence of suicide between the individuals with and without depression, with exceptionally high risks among the adults over 65 years and college graduates. These findings underscore the complex interplay between mental health, societal pressures, and age-related challenges in South Korea. Older adults often face social isolation, economic insecurity, and weakened family support, while young college graduates grapple with intense academic and career expectations in a competitive job market [48]. People are increasingly becoming discouraged from seeking help for mental health issues due to the stigma associated with them, which increases the likelihood of severe consequences such as suicide [12].

Despite providing valuable insights, this study has several significant limitations that should be acknowledged. The cross-sectional design limits the capacity to demonstrate a causal relationship between depressive symptoms and sociodemographic variables, enabling merely the observation of correlations. The reliance on retrospective self-reports also introduces potential biases, such as recall and reporting biases, which may lead to underreporting of depressive symptoms due to stigma. Future research should employ longitudinal designs and gather corroborative information from multiple sources, including family members, to confirm causal relationships and minimize bias. Despite these limitations, this study has strengths including being the first to differentiate prevalence trends in subthreshold and major depression across sociodemographic subgroups within South Korea’s adult population.

## Conclusions

Based on the study findings, South Korea experienced a significant increase in the prevalence of both subthreshold and major depression from 2014 to 2018, with notable variations across different sociodemographic groups. The prevalence of depression increased more sharply among the women, high school and college graduates, and individuals who frequently consumed alcohol. This trend is likely influenced by South Korea’s rapid socioeconomic changes, intense academic and career pressures, persistent gender inequalities, and cultural norms surrounding alcohol use. The study also highlighted major depression, age over 65 years, and college education as high-risk factors for suicide attempts. These findings underscore the complex interplay between mental health, societal pressures, and demographic shifts in South Korea, emphasizing the need for targeted interventions and policies to address the growing mental health challenges faced by various segments of the population.
